# pCO_2_ and pH regulation of cerebral blood flow

**DOI:** 10.3389/fphys.2012.00365

**Published:** 2012-09-14

**Authors:** SeongHun Yoon, Mario Zuccarello, Robert M. Rapoport

**Affiliations:** ^1^Research Service, Department of Pharmacology and Cell Biophysics, Veterans Affairs Medical Center, University of Cincinnati College of MedicineCincinnati, OH, USA; ^2^Surgical Service, Department of Neurosurgery, Veterans Affairs Medical Center, The Neuroscience Institute, University of Cincinnati College of MedicineCincinnati, OH, USA

**Keywords:** carbon dioxide, cerebral blood flow, nitric oxide, respiratory acidification, respiratory alkalinization, vasoactive factors

## Abstract

CO_2_ serves as one of the fundamental regulators of cerebral blood flow (CBF). It is widely considered that this regulation occurs through pCO_2_-driven changes in pH of the cerebral spinal fluid (CSF), with elevated and lowered pH causing direct relaxation and contraction of the smooth muscle, respectively. However, some findings also suggest that pCO_2_ acts independently of and/or in conjunction with altered pH. This action may be due to a direct effect of CSF pCO_2_ on the smooth muscle as well as on the endothelium, nerves, and astrocytes. Findings may also point to an action of arterial pCO_2_ on the endothelium to regulate smooth muscle contractility. Thus, the effects of pH and pCO_2_ may be influenced by the absence/presence of different cell types in the various experimental preparations. Results may also be influenced by experimental parameters including myogenic tone as well as solutions containing significantly altered HCO_3_^−^ concentrations, i.e., solutions routinely employed to differentiate the effects of pH from pCO_2_. In sum, it appears that pCO_2_, independently and in conjunction with pH, may regulate CBF.

## Relaxation/contraction

### pH-dependent

A major physiologic regulator of cerebral blood flow (CBF) is arterial blood pCO_2_, with hyper- and hypo- capnia resulting in dilatation and contraction of the cerebral vasculature, respectively (Harper and Bell, 1963). The mechanism underlying this regulation appears independent of the decreased and increased arterial pH levels accompanying the elevated and lowered pCO_2_, respectively, since CBF remains unchanged following metabolic acidosis and alkalosis (Lambertsen et al., 1961; Harper and Bell, 1963; Severinghaus and Lassen, 1967; Nau et al., 1999; Anderson and Meyer, 2002). Rather, findings suggest that CBF is regulated by changes in pH of the cerebral spinal fluid (CSF) as the result of the rapid equilibration between CO_2_ in the arterial blood and CSF (Lambertsen et al., 1961; Harper and Bell, 1963; Severinghaus and Lassen, 1967; Lassen, 1968). The lowered/elevated pH in the CSF then acts directly on the vasculature to cause relaxation and contraction, respectively (Lambertsen et al., 1961; Harper and Bell, 1963; Severinghaus and Lassen, 1967; Lassen, 1968). Thus, the action of pCO_2_ on the vasculature is restricted to that of altering CSF pH, i.e., is void of other indirect effects as well as direct effects (Lambertsen et al., 1961; Harper and Bell, 1963; Severinghaus and Lassen, 1967; Lassen, 1968).

Evidence in support of this proposal includes demonstrations in cat and rat cranial window preparations that acidic hypercapnic but not isohydric hypercapnic superfusate caused pial arteriolar dilatation (Wahl et al., 1970; Kontos et al., 1977). Acidic hypercapnic but not isohydric hypercapnic solutions also dilated precapillary microvessels in rat cerebral cortex brain slices (Nakahata et al., 2003). Similarly, alkaline hypocapnic but not isohydric hypocapnic superfusate contracted cat and rat pial arterioles in cranial window preparations (Wahl et al., 1970; Kontos et al., 1977) and pressurized segments of rat penetrating cerebral arterioles (Apkon and Boron, 1995).

Additionally, the magnitude of the dilatation/contraction in response to solutions with altered pH are similar in the absence and presence of corresponding changes in pCO_2_. For example, in a cranial window in the cat, acidic hypercapnic and acidic isocapnic superfusate elicited similar magnitudes of dilatation of pial arterioles (Kontos et al., 1977). Also, similar magnitudes of dilatation following challenge with acidic hypercapnic and acidic isocapnic solutions were observed in several *in vitro* vascular preparations from the rat, including basilar and middle cerebral artery rings and pressurized segments of middle cerebral artery and parenchymal vessels (You et al., 1994; Tian et al., 1995; Peng et al., 1998a,b; Dabertrand et al., 2012). Alkaline hypocapnic and alkaline isocapnic solutions also elicited similar magnitudes of contraction of pial arterioles in a cranial window in the cat (Kontos et al., 1977) and in rings of rabbit basilar artery (Zuccarello et al., 2000a,c).

General support for the hypothesis that extracellular pH rather than pCO_2_ alters cerebrovascular contractility can also be derived from findings that acidic and alkaline solutions in the absence of changes in pCO_2_ cause dilatation and contraction, respectively. Specifically, acidic isocapnic solution infused ventriculocisternally increased total and/or regional CBF in dog (Siesjö et al., 1968; Pannier et al., 1972; Britton et al., 1979; Koehler and Traystman, 1982) and superfused in a cranial window increased CBF (Wang et al., 1992) and dilated pial arterioles in rat (Xu et al., 2004). Also, *in vitro* perfusion with acidic isocapnic solution dilated pressurized segments of rat middle cerebral artery and penetrating cerebral arterioles (Dacey and Duling, 1982; Dietrich and Dacey, 1994; Dietrich et al., 1994; Lindauer et al., 2001; Horiuchi et al., 2002).

Similarly, alkaline isocapnic solution infused ventriculocisternally decreased total and/or regional CBF in dog (Pannier et al., 1972; Britton et al., 1979; Koehler and Traystman, 1982) and superfused in a cranial window decreased CBF in rat cortex (Liu et al., 2012) and contracted cat pial arterioles (Kontos et al., 1977). Alkaline isocapnic superfusate, albeit unexpectedly not alkaline hypocapnic superfusate, also contracted rabbit basilar artery (Yoon et al., 2002a, 2003). Additionally, *in vitro* perfusion with alkaline isocapnic solution contracted pressurized segments of rat penetrating cerebral arterioles and middle cerebral artery (Dacey and Duling, 1982; Smeda et al., 1987; Dietrich and Dacey, 1994; Dietrich et al., 1994; Lindauer et al., 2001; Horiuchi et al., 2002), and isolated smooth muscle cells from guinea pig basilar artery (West et al., 1992).

Also in support of local extracellular pH and not pCO_2_ as the major determinant of vascular contractility in response to respiratory hypercapnia/hypocapnia is the ability of alkaline isocapnic superfusate to markedly reduce the increased CBF due to respiratory hypercapnia in rat cerebral cortex (Liu et al., 2012). Respiratory hypercapnia-induced increased regional CBF was also greatly decreased following ventriculocisternal infusion of pH 7.52 solution containing 60 mM HCO_3_^−^ in the dog (Koehler and Traystman, 1982). Consistent with the overall premise that local extracellular pH and/or pCO_2_ is the major determinant of vascular contractility is the conclusion that alkaline hypocapnic superfusate completely prevented respiratory hypercapnia-induced pial vessel dilatation in the cat (Kontos et al., 1977). On the other hand, after taking into account the decrease in basal pial vessel diameter due to the alkaline hypocapnic superfusate, the ~27% pial dilatation due to respiratory hypercapnia was only reduced to ~18% (Kontos et al., 1977).

### pCO_2_-dependent

Studies in both isolated and *in situ* vascular preparations suggest that pCO_2_ independently of pH can regulate CBF. In isolated ring segments of cat middle cerebral artery, presumably with intact endothelium, significant contraction was induced by lowering pCO_2_ in the bathing solution from 37 to 14 mmHg while maintaining pH at 7.4 (Harder and Madden, 1985). Furthermore, although pH 7.6/pCO_2_ 14 mmHg solution further increased contraction, relaxation was induced upon subsequent elevation of pCO_2_ to 37 mmHg while pH was maintained at 7.6 (Harder and Madden, 1985).

Also, while acidic hypercapnic solution dilated endothelium intact helical strips of dog basilar and middle cerebral artery contracted with 20 mM KCl, return to normal pH through elevation of HCO_3_^−^ to 75 mM in the continued presence of elevated pCO_2_ resulted in initial re-constriction to control levels followed by partial relaxation (Toda et al., 1989). These findings (Toda et al., 1989) suggest that elevated pCO_2_ levels in the absence of a change in pH can cause relaxation.

In other vascular preparations, elevated pH appears insufficient to elicit contraction (Edvinsson and Sercombe, 1976; Aoyama et al., 1999; Kim et al., 2004). Specifically, in rabbit basilar artery ring/strip preparations elevation of extracellular pH to 7.9 with unchanged CO_2_ did not cause contraction (Aoyama et al., 1999; Kim et al., 2004). Whether the lack of contraction in response to elevated pH was the result of nitric oxide synthase (NOS) inhibition by N^ω^-nitro-L-arginine (L-NNA; Kim et al., 2004) or absence of the endothelium (Aoyama et al., 1999) should be considered. On the other hand, in cat middle cerebral artery rings presumably with intact endothelium, elevation of extracellular pH only occasionally induced contraction and, even then, the contraction was of small magnitude (Edvinsson and Sercombe, 1976).

There is also some evidence from our laboratory which suggests that pH as well as pCO_2_ in the CSF compartment do not serve as the major regulators of respiratory hypocapnia-induced vasoconstriction (Yoon et al., 2002b). Using a cranial window preparation in the rabbit (with underlying ketamine/xylazine-induced acute metabolic alkalosis) we demonstrated that the magnitude of contraction of the basilar artery to lowered pCO_2_ as the result of increased respiration was similar with superfusates at pH 7.3/49.0 mmHg pCO_2_ (gassed with 8% CO_2_) and pH 7.5/30.6 mmHg pCO_2_ (gassed with 5% CO_2_; Yoon et al., 2002b).

**Figure 1:**
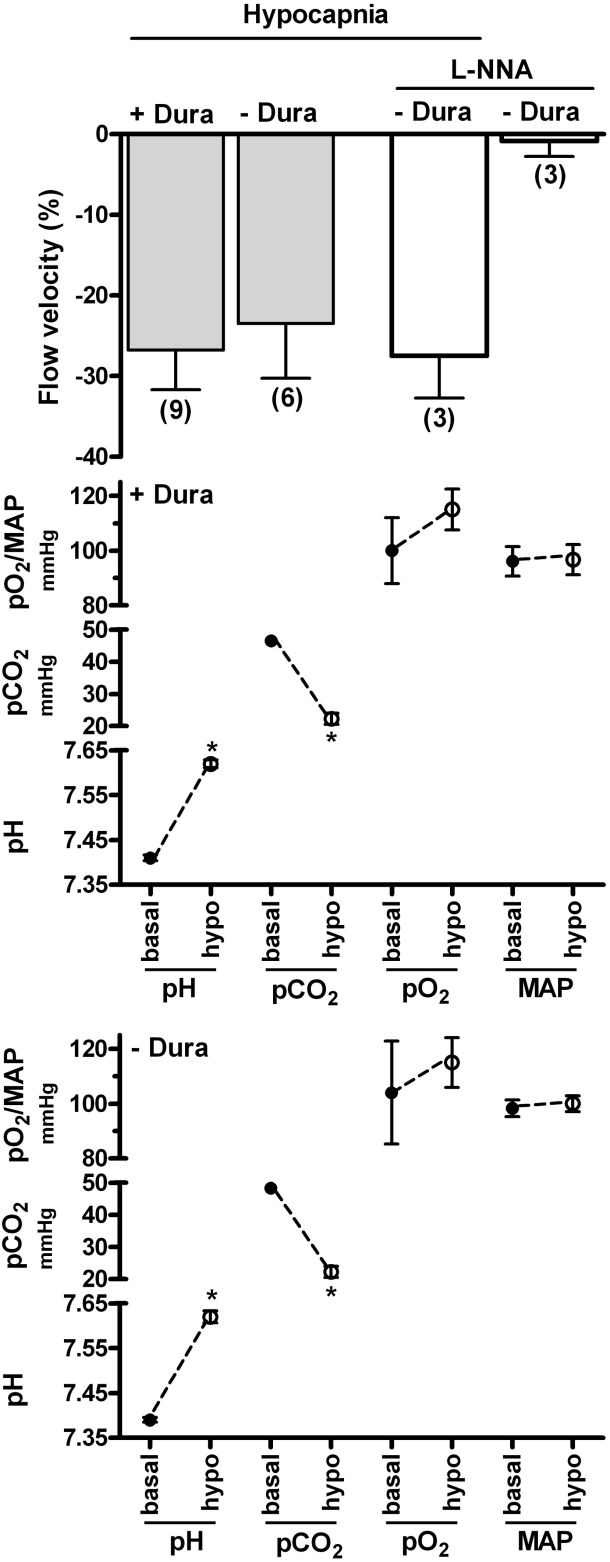
**Effects of respiratory hypocapnia in the presence and absence of superfusate and of NO synthase inhibitor on blood flow velocity in rat ventral midbrain.** Respiratory rate and/or tidal volume were increased in cranial window preparations superfused with Krebs-Ringer bicarbonate solution (−Dura) or in preparations with a burr hole in the cranium (+Dura). Laser-Doppler flow (flow velocity) was calculated as percent baseline (top panel). Arterial blood gas parameters and mean arterial pressure (MAP) are indicated in the lower panels and were similar in preparations with and without dura. Additional superfused preparations were exposed to 0.1 mM L-NNA for 30 min prior to respiratory hypocapnic challenge.

Additionally, we compared laser-Doppler CBF in rat ventral midbrain in response to respiratory hypocapnia in a cranial window preparation superfused with pH 7.4 Krebs-Ringer bicarbonate solution and in a preparation which contained only a burr hole in the cranium. Thus, in the superfused preparation the extracellular pH/pCO_2_ were constant while in the burr hole preparation the CSF pCO_2_ was allowed to decrease, thereby elevating pH. This experimental protocol retains the advantage that the HCO_3_^−^ concentration in the superfusate is maintained at a physiologic level, thereby eliminating possible artifact due to elevated HCO_3_^−^ concentration (Yoon et al., 2003; see “Physiologic and non-physiologic parameters”). Increased respiration, which lowered arterial pCO_2_ levels to similar magnitudes in both the superfused and burr hole preparations (from ~47 mmHg to ~22 mmHg), induced similar magnitudes of decreased CBF (Figure 1). Thus, the combined observations of the apparent lack of effect of extracellular (CSF) pH/pCO_2_ on the contraction/decreased CBF due to lowered pCO_2_ by increased respiration (Yoon et al., 2002b; Figure 1), along with the lack of effect of metabolic alkalosis on CBF (Nau et al., 1999; Anderson and Meyer, 2002), may suggest that pCO_2_ also regulates cerebrovascular contractility through an action within the arterial blood compartment.

**Figure 2:**
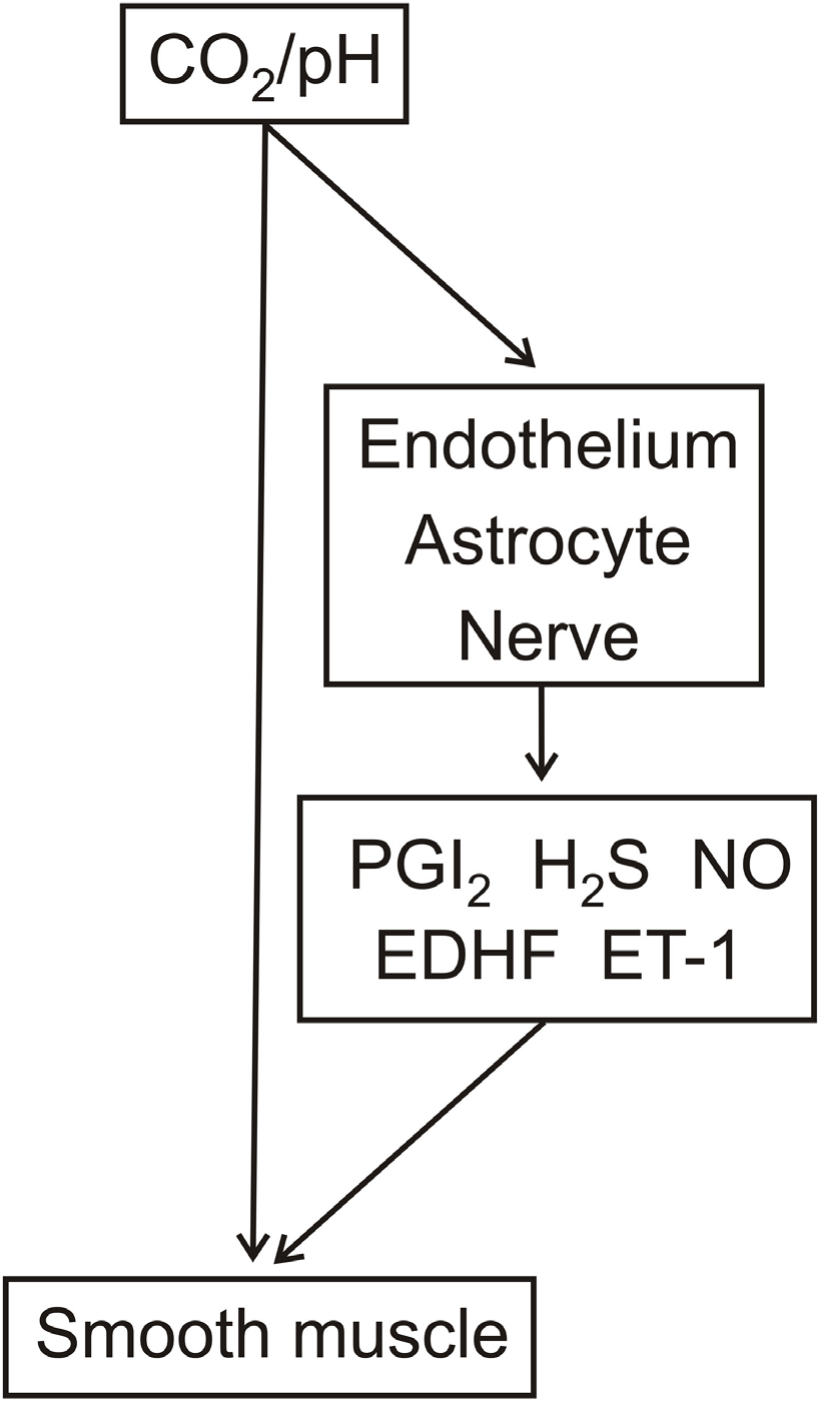
**Potential cellular sites of action and vasoactive factor involvement in respiratory hypercapnia/hypocapnia-mediated relaxation/contraction.** The effects of respiratory hypercapnia/hypocapnia may be mediated through accompanying changes in pH and/or directly by pCO_2_. pH and/or pCO_2_ act on the smooth muscle as well as on the endothelium, nerves, and astrocytes. These latter cell types release vasoactive factors, including PGI_2_, H_2_S, NO, endothelium-derived hyperpolarizing factor (EDHF), and endothelin-1 (ET-1).

It is of interest to speculate that this regulation could occur through an action of arterial pCO_2_ on the endothelium. Indeed, a possible mechanism underlying such a regulatory pathway may reflect the following: (1) increased pCO_2_, but not lowered extracellular pH, decreased intracellular pH and elevated intracellular Ca^2+^ in cultured rat aorta endothelial cells (Ziegelstein et al., 1993) and (2) inhibition of endothelial SK_Ca_/IK_Ca_ channels resulted in contraction of pressurized parenchymal vessels in rat and also reduced laser-Doppler CBF in mice (Hannah et al., 2011). Thus, the increased/decreased endothelial intracellular Ca^2+^ levels could regulate smooth muscle contractility through changes in smooth muscle membrane potential as determined by negative charge transfer via myo-endothelial gap junctions and/or release of K^+^ (Sandow et al., 2009; Edwards et al., 2010). It is also of interest to note that the pathway responsible for the release of prostaglandin I_2_ and H_2_S, which were suggested to mediate respiratory hypercapnia-induced dilatation of pial vessels in the newborn pig, was schematically illustrated to result from the lowering of intracellular pH in endothelial cells by elevated CO_2_ (Leffler et al., 2011). Thus, as illustrated in Figure 2, CO_2_ modulation of EDHF release as well as endothelial release of prostaglandin I_2_ and H_2_S may represent pathways for the regulation of vascular contractility.

Clearly, an endothelial action of pCO_2_ to regulate contractility through these and possibly other endothelial derived vasoactive factors (see below) necessitates the presence of a functional endothelium. Consistent with this requirement is the demonstration that localized light-dye endothelial injury abolished respiratory hypercapnia-induced dilatation in pial arterioles of newborn and juvenile pigs (Willis and Leffler, 2001). On the other hand, respiratory hypercapnia-induced dilatation of rat pial arterioles was not inhibited following localized light-dye injury of the endothelium (Wang et al., 1994). Whether these contrasting findings of endothelial dependency (Wang et al., 1994; Willis and Leffler, 2001) is related to different animal age and/or species remains a possibility.

### NO

Direct evidence for CO_2_ modulation of NO release was recently demonstrated by isohydric hypercapnic- and isohydric hypocapnic solution-induced increased and decreased NO release, respectively, from both cultured endothelium from adult human temporal lobe microvessels and astrocytes from human fetus (Fathi et al., 2011). Also consistent with the pCO_2_ rather than pH regulation of NO release is the demonstration that a neuronal NOS inhibitor partially prevented respiratory hypercapnia-induced dilatation of rat pial arterioles while dilatation due to acidic isocapnic superfusate remained unaltered (Xu et al., 2004). Thus, as illustrated in Figure 2, CO_2_ modulation of NO release from the endothelium, astrocytes, and neurons may represent additional pathways for the regulation of vascular contractility.

The relevance of the decreased NO release due to lowered pCO_2_ (Fathi et al., 2011), however, is not readily apparent since the time course for the decreased release was delayed as compared to the rapid inhibitory effect of systemic NOS inhibitor on the respiratory hypercapnia-elevated cortical CBF in, e.g., primates (Thompson et al., 1996). Thus, it was suggested that the modulation of NO release in response to pCO_2_ is related to non-acute changes in contractility (Fathi et al., 2011). It is also of interest to speculate that CO_2_ may modulate NO release from cellular sites other than endothelium and astrocytes, such as neurons (Xu et al., 2004; reviewed in Toda et al., 2009).

The relationship between the decreased NO release by isohydric hypocapnic solution (Fathi et al., 2011) and respiratory hypocapnia-induced cerebral vasoconstriction is also not clear since NOS inhibitors did not reduce the contraction elicited by respiration-induced lowered pCO_2_ in the rabbit (Toda et al., 1989) and rat basilar artery *in situ* (21.8% and 22.3% contraction in the absence and presence of L-NNA, respectively; SH Yoon, M Zuccarello, RM Rapoport, unpublished observation). L-NNA Also did not reduce the decreased laser-Doppler flow in rat ventral midbrain due to lowered pCO_2_ (Figure 1), even though L-NNA contracted the rat basilar artery *in situ* by ~10% (SH Yoon, M Zuccarello, RM Rapoport, unpublished observation).

Although L-NNA did not decrease basal laser-Doppler flow in rat ventral midbrain (Figure 1), this lack of effect could reflect regional differences, as previously observed following systemic administration of NOS inhibitor in the cat (Kovách et al., 1992). Indeed, L-NNA superfusion decreased basal laser-Doppler CBF in rat cerebral cortex (Iadecola and Xu, 1994; Iadecola and Zhang, 1996), while not in cerebellum (Fabricius and Lauritzen, 1994). However, in possible contrast to the decreased basal flow in rat cerebral cortex (Iadecola and Xu, 1994; Iadecola and Zhang, 1996), basal CBF was not decreased in rat parietal cortex (Fabricius and Lauritzen, 1994).

### Endothelin-1

Evidence for a role of endothelin (ET)-1 in pCO_2_- or, for that matter, pH-mediated changes in contractility is sparse. We demonstrated in rabbit basilar artery *in situ* that contraction of the rabbit basilar artery to lowered pCO_2_ by increased respiration as well as to isocapnic alkaline superfusate was relaxed by ET receptor antagonists (Yoon et al., 2002a). Additionally, pretreatment with an ET converting enzyme inhibitor decreased the contraction of the rabbit basilar artery to lowered pCO_2_ by 50%, although challenge with the ET converting enzyme inhibitor during the plateau contraction resulted in only 20% relaxation, while relaxing the contraction to isocapnic alkaline superfusate by 75% (Yoon et al., 2002a). We also demonstrated that ET receptor antagonists relaxed contraction to hypocapnic- and isocapnic-alkaline solution in rabbit basilar artery *in vitro* (Zuccarello et al., 2000a,b,c).

Although, elevated levels of arterial ET-1 were not observed in humans following hyperventilation for 4 min or for repeated 2 min intervals (Jordan et al., 2002; Peebles et al., 2008), alkaline hypocapnic solution increased ET-1 release from pig cerebral endothelial cells in culture (Yoshimoto et al., 1991). However, since the effect of isohydric hypocapnic solution on ET-1 release was not investigated, it remains unclear whether the alkaline pH and/or lowered pCO_2_ were responsible for the increased ET-1 release (Yoshimoto et al., 1991). Additionally, based on the ability of lowered pCO_2_ to decrease NO release (Fathi et al., 2011), it may be considered that respiratory hypocapnia-induced increased ET-1 release is secondary to disinhibition of decreased ET-1 release by NO (Boulanger and Luscher, 1990). On the other hand, this ET-1 release mechanism would not be present in the rabbit basilar artery since L-NNA did not inhibit contraction to respiration-induced lowered pCO_2_ (Yoon et al., 2002b). Pathways which depict the pCO_2_/pH regulation of ET-1 release are also represented in Figure 2.

### 20-hydroxyicosatetraenoic acid

The role of 20-hydroxyicosatetraenoic acid (20-HETE) in the CO_2_/pH regulation of contractility was investigated in pial arterioles of fetal sheep *in situ* (Ohata et al., 2010). Superfusion with the omega-hydroxylase inhibitor, 17-octadecynoic acid, did not prevent contraction of the pial vessels to respiratory hypocapnia (Ohata et al., 2010). Thus, it does not appear, at least based on this study (Ohata et al., 2010), that 20-HETE is involved in the CO_2_/pH regulation of cerebral vascular contractility.

## Signaling pathways in smooth muscle

An action of CO_2_ independent of or concomitant with that of pH was also demonstrated by the greater increase in inositol trisphosphate levels by alkaline hypocapnic than alkaline isocapnic solution in cultured cerebral microvascular smooth muscle cells from piglets (Albuquerque et al., 1995). Additionally, in rat middle cerebral artery rings and pressurized segments, while both acidic hypercapnic and acidic isocapnic solutions induced relaxation, these solutions elicited membrane hyperpolarization and depolarization, respectively (Peng et al., 1998a,b). Also, acidic isocapnic and essentially isohydric hypercapnic solution were without effect and hyperpolarized rat middle cerebral artery muscle cells, respectively (Harder, 1982).

## Physiologic and non-physiologic parameters

Some of the varied effects of pCO_2_ and pH on cerebrovascular contractility described above could be attributed to vessel location within the cerebrovascular tree as well as animal age and species. This possibility was previously described with respect to the involvement of K_ATP_ channels in the pH-mediated relaxation/contraction of cerebral vessels (Rosenblum, 2003). In addition, experimental parameters or inherent differences between preparation types may also result in ambiguity with regards to pCO_2_- and pH-dependent mechanisms, as follows:

1. Non-physiologic pH: Hypercapnia and hypocapnia can lower and elevate CSF pH by ~0.3 pH units, i.e., to ~7.1 and ~7.6, respectively (Plum and Posner, 1967; Adaro et al., 1969; Nilsson and Busto, 1973). Thus, non-physiologic mechanisms may be elicited by solutions with pH values outside of this range. For example, reversible contraction was elicited by pH 7.8 but not pH 8.0 Hepes buffered solution in isolated perfused penetrating arterioles of the rat middle cerebral artery (Apkon and Boron, 1995).
2. Absence of HCO_3_^−^: The lack of inclusion of HCO_3_^−^ may produce artifacts since HCO_3_^−^, through the Na^+^ dependent Cl^−^-HCO_3_^−^ exchanger, is required to recover from an alkali load and for normal cell homeostasis (Kikeri et al., 1990; Neylon et al., 1990; Batlle et al., 1993). The importance of HCO_3_^−^ was also shown by the amplification of changes in intracellular pH when intraluminally pressurized rat cerebral arterioles were exposed to alkaline hypocapnic solution as compared to alkalinized Hepes solution (Apkon and Boron, 1995). Moreover, we demonstrated that alkaline Tris buffered solution but not alkaline hypocapnic Krebs-Ringer bicarbonate solution activated extracellular regulated protein kinase in brain microvascular endothelial cells (Motz et al., 2006).
3. Alkaline isocapnic solution: A standard procedure for the evaluation of the effects of alkaline pH in the absence of changes in CO_2_ is through the use of alkaline isocapnic solution, attained through the elevation of the HCO_3_^−^ concentration. However, we observed that even relatively modest elevation of the HCO_3_^−^ concentration from 25 mM to 35 mM or to 50 mM, yielding alkaline isocapnic superfusate with pH elevated from 7.4 to 7.6 and to 7.7, respectively, desensitized the contractile response of the rabbit basilar artery *in situ* to subsequent challenge with these alkaline isocapnic superfusates and to respiratory hypocapnia (Yoon et al., 2003). It should be noted, however, that the findings of desensitization (Yoon et al., 2003) may be complicated by the underlying anesthetic (ketamine/xylazine)-induced acute metabolic alkalosis (Yoon et al., 2002b). Moreover, unexpectedly, alkaline hypocapnic superfusate failed to contract the rabbit basilar artery (Yoon et al., 2002a).
  Despite these potential complications, the desensitization of contraction to alkaline isocapnic superfusate and respiratory hypocapnia by prior alkaline isocapnic superfusate may suggest that elevated HCO_3_^−^ concentration elicits additional cellular events not present during acute changes in pCO_2_. Indeed, the calculated HCO_3_^−^ concentration in CSF after 1–2 h of respiratory hypercapnia and hypocapnia was not significantly altered in human and only small changes were observed in dog and cat (Paddle and Semple, 1969 and references therein; Shibata et al., 1976; Kazemi and Javaheri, 1978). For example, in dog even after 2 h of respiratory hypercapnia, which increased CSF pCO_2_ from 46.7 to 80 mmHg, the calculated HCO_3_^−^ concentration increased by only 3.3 mM (Shibata et al., 1976). Also in dog, even after 2 h of respiratory hypocapnia, which decreased CSF pCO_2_ from 48 to 30 mmHg, the calculated HCO_3_^−^ concentration decreased by only 4.9 mM (Kazemi and Javaheri, 1978).
4. Levels of pCO_2_/pH: Different mechanisms of dilatation/contraction may be evoked depending on the level of pCO_2_/pH. For example, the K_ATP_ channel blocker, glyburide, inhibited dilatation to moderate but not severe hypercapnia in arterioles of the rabbit cerebral cortex (Faraci et al., 1994). Also, NOS inhibition decreased laser-Doppler CBF to moderate and severe but not to extreme hypercapnia in rat cerebral cortex (Iadecola and Zhang, 1994). Possibly consistent with the suggestion that the contraction due to respiratory hypocapnia involves multiple mechanisms is our demonstration in rabbit basilar artery *in situ* that alkaline isocapnic superfusate with elevated HCO_3_^−^ concentration only partially inhibited subsequent contraction to respiratory-induced lowered pCO_2_ (Yoon et al., 2003). Moreover, the magnitude of decreased contraction to respiratory-induced lowered pCO_2_ was similar to the decreased contraction to a second challenge with isocapnic alkaline superfusate (Yoon et al., 2003). Thus, the contraction due to respiration-induced lowered pCO_2_ may be composed of components sensitive and insensitive to desensitization by alkaline isocapnic solution (Yoon et al., 2003). It should also be considered that the mechanism underlying contraction to pCO_2_/pH may differ depending on the time post initial alteration in pCO_2_/pH.
5. *In vitro* vs. *in vivo*: We demonstrated in rabbit basilar artery rings *in vitro* that maintained contraction to alkaline hypocapnic and isocapnic solutions was infrequently initiated in the absence of initial triggering with repeated challenge with KCl, serotonin or NOS inhibitor (Zuccarello et al., 2000a,b,c). Possibly consistent with these findings (Zuccarello et al., 2000a,b,c) are observations in rabbit basilar and cat middle cerebral artery ring/strip preparations in which elevation of extracellular pH did not cause or elicited only minimal contraction (Edvinsson and Sercombe, 1976; Aoyama et al., 1999; Kim et al., 2004). Additionally, contraction of cat middle cerebral artery rings to pH 7.4/pCO_2_ 14 mmHg solution was highly variable and, in some cases, minimal (Harder and Madden, 1985). Thus, factors may be absent/altered in *in vitro* ring/strip preparations that are present *in vivo*.
  It is of interest to speculate that intraluminal pressure is a factor which enhances alkaline hypocapnic solution-induced contraction *in vivo* and is absent from *in vitro* ring/strip preparations. Indeed, smooth muscle depolarization associated with intraluminal pressure (Dunn et al., 1994) could influence the actions of vasoactive factors as well as direct effects of CO_2_/pH on the smooth muscle. Additionally, with respect to contractile/relaxant mechanism, the absence of intraluminal pressure can completely change the mechanism whereby endothelium-derived factors elicit changes in contractility. For example, in small arteries of mouse gracilis muscle the absence of intraluminal pressure abolished the ability of acetylcholine to induce relaxation through endothelium-derived hyperpolarizing factor (Boettcher and de Wit, 2011).
  Also, differing basal neuronal and endothelial NO release in *in vivo* vs. *in vitro* preparations may influence findings with respect to NO-dependency (Toda et al., 1996).
6. Absence of cell types: Depending on the vessel type/location, findings in isolated vessels could be altered by the absence of astrocytes and lack of nerve input since these cells have been implicated in changes in contractility in response to altered CO_2_ (Xu et al., 2004; Toda et al., 2009; Fathi et al., 2011). For example, damage of the glial limitans with L-α-aminoadipic acid resulted in partial inhibition of respiratory hypercapnia-induced dilatation of rat pial arterioles while not altering dilatation to acidic isocapnic superfusate (Xu et al., 2004). Thus, these findings suggest the involvement of glial and neuronal cells in pCO_2_ regulation of CBF (Xu et al., 2004). This involvement appears to be age and possibly species dependent since L-α-aminoadipic acid failed to inhibit respiratory hypercapnia-induced dilatation of pial arterioles in newborn pig (Parfenova et al., 2012).

## Conclusion

pCO_2_ and pH may independently and concomitantly regulate cerebrovascular contractility. While regulation by pH appears restricted to the CSF, regulation by pCO_2_ may occur through an action within the CSF and also possibly within the arterial blood. This latter regulation could occur through endothelium-dependent mechanisms. Additional sites of pCO_2_ action, as well of pH, may include nerves and astrocytes. The possible effects of pCO_2_ and pH on these different cell types and the involvement of vasoactive factors are outlined in Figure 2.

The underlying pCO_2_/pH regulatory mechanism(s) also likely varies with vessel type and magnitude and duration of challenge with altered pCO_2_/pH. Furthermore, these mechanisms may reflect redundant pathways (Irikura et al., 1995; Wang et al., 1998; Xu et al., 2004; Fathi et al., 2011; Leffler et al., 2011). Finally, with respect to experimental variables, the preparation and conditions employed likely influence the observed mechanism. Overall, the numerous and interactive regulatory pathways provides precise, regional regulation of CBF by pCO_2_ and pH (Ito et al., 2000; Willie et al., 2012).

### Conflict of interest statement

The authors declare that the research was conducted in the absence of any commercial or financial relationships that could be construed as a potential conflict of interest.
